# Behavioral characterization of the 6-hydroxidopamine model of Parkinson’s disease and pharmacological rescuing of non-motor deficits

**DOI:** 10.1186/1750-1326-8-14

**Published:** 2013-04-26

**Authors:** Miguel M Carvalho, Filipa L Campos, Bárbara Coimbra, José M Pêgo, Carla Rodrigues, Rui Lima, Ana J Rodrigues, Nuno Sousa, António J Salgado

**Affiliations:** 1 Life and Health Sciences Research Institute (ICVS), School of Health Sciences, University of Minho, Campus de Gualtar, Braga, Portugal; 2 ICVS/3B’s, PT Government Associate Laboratory, Braga/Guimarães, Portugal

**Keywords:** Parkinson’s disease, 6-OHDA, Motor behavior, Emotion, Levodopa, Bupropion, Paroxetine

## Abstract

**Background:**

Parkinson’s disease (PD) is a chronic neurodegenerative condition that is characterized by motor symptoms as a result of dopaminergic degeneration, particularly in the mesostriatal pathway. However, in recent years, a greater number of clinical studies have focused on the emergence of non-motor symptoms in PD patients, as a consequence of damage on the mesolimbic and mesocortical dopaminergic networks, and on their significant impact on the quality of life of PD patients. Herein, we performed a thorough behavioral analysis including motor, emotional and cognitive dimensions, of the unilateral medial forebrain bundle (MFB) 6-hydroxidopamine (6-OHDA)-lesioned model of PD, and further addressed the impact of pharmacological interventions with levodopa and antidepressants on mood dimensions.

**Results:**

Based on apomorphine-induced turning behaviour and degree of dopaminergic degeneration, animals submitted to MFB lesions were subdivided in complete and incomplete lesion groups. Importantly, this division also translated into a different severity of motor and exploratory impairments and depressive-like symptoms; in contrast, no deficits in anxiety-like and cognitive behaviors were found in MFB-lesioned animals. Subsequently, we found that the exploratory and the anhedonic behavioural alterations of MFB-lesioned rats can be partially improved with the administration of both levodopa or the antidepressant bupropion, but not paroxetine.

**Conclusions:**

Our results suggest that this model is a relevant tool to study the pathophysiology of motor and non-motor symptoms of PD. In addition, the present data shows that pharmacological interventions modulating dopaminergic transmission are also relevant to revert the non-motor behavioral deficits found in the disease.

## Background

Parkinson’s disease (PD) is a complex neurodegenerative chronic disease affecting the dopaminergic system. First described by James Parkinson in 1817 [1], this disease affects mainly the *substantia nigra pars compacta* (SNc), also designated as area A9, where a massive dopaminergic degeneration takes place [2-4]. This brain area is the origin of the mesostriatal dopaminergic projections to the *caudate/putamen* complex [5]. Thus, degeneration in this pathway causes functional disruption of basal ganglia’s circuitry [6], and the emergence of several physical hallmarks of PD, such as bradykinesia and tremors [7]. Though to a lesser extent, the mesocorticolimbic dopaminergic pathway, originated in area A10, is also affected as the disease progresses [8-11]. This event will lead to the development of several non-motor features of PD including depression, apathy, anhedonia, and dementia [8,12-14]. Although it was somewhat disregarded in the past, more recent evidence has shown that mood and cognitive changes associated with PD have a clear effect on the quality of life of the patients [15,16].

The development of animal models of PD, using both genetic and toxin-based approaches, was of great importance to the field, as it improved our knowledge on the pathophysiological mechanisms of the disease and provided experimental tools for testing novel therapies. Among PD models, the unilaterally 6-hydroxidopamine (6-OHDA)-lesioned rat is one of particular interest [17,18]. The lesion protocol, usually done in the medial forebrain bundle (MFB), is known to cause significant motor deficits; and given its unilateral nature, it causes an inter-hemispheric imbalance in dopamine (DA) transmission that permits the assessment of a quantifiable turning behavior, which can be correlated with the extension of the lesion [19-23]. However, since PD is known to have significant impact in mood and cognition, it is of the utmost importance to identify and characterize additional behavioral features besides motor disabilities, in order to fully understand the potential of this model in the context of PD. For this purpose, we performed herein a detailed motor and non-motor behavioral characterization of the unilateral MFB-lesioned PD model. In addition, to further explore the relevance of combined therapeutic approaches for PD, in which both motor and non-motor symptoms are considered, we tested the ability of levodopa (LD), the selective serotonin reuptake inhibitor (SSRI) paroxetine (PRX) and the norepinephrine/dopamine reuptake inhibitor bupropion (BUP), to revert some of the deficits displayed in cases of severe PD-like lesions.

We show that 6-OHDA MFB injections caused a variable level of dopaminergic degeneration in both mesostriatal and mesocorticolimbic pathways, and significantly contributed for the development of motor and specific non-motor impairments. Furthermore, we showed that the pharmacological manipulation of the dopaminergic (and noradrenergic) system is able to revert some of the motivational/hedonic deficits found in cases of severe MFB lesions.

## Results

### Phenotypic and histological characterization of 6-OHDA lesions

**Figure 1:**
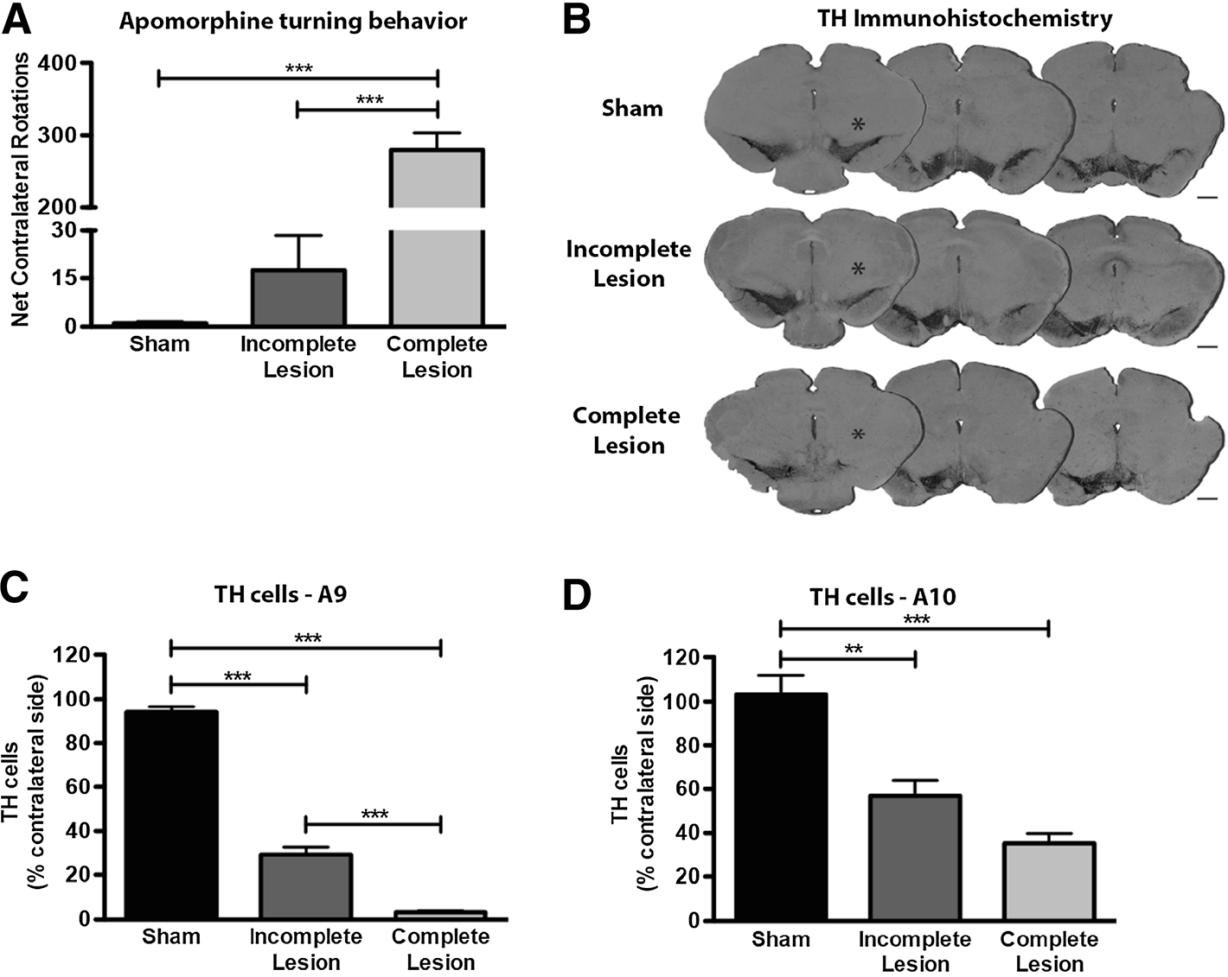
**Characterization and quantification of 6-OHDA induced lesions. A**) Apomorphine-induced turning behavior revealed two classes of 6-OHDA lesioned animals, those with reduced turning behavior (incomplete lesion) and those with intense turning behavior (complete lesion). Sham animals showed no turning behavior. **B**) Representative photomicrographs of brain slices stained for TH. Compared to sham group, both incomplete and complete lesion groups display marked reduction of TH staining in areas A9 and A10 of the lesioned hemisphere (*). Scale bar = 1 mm. **C**) Quantification of lesion extent in area A9 shows a ~70% reduction of TH staining in the incomplete lesion group and a ~97% reduction in the complete lesion group. **D**) Quantification of lesion extent in area A10 shows a ~40% reduction of TH staining in the incomplete lesion group and a ~60% reduction in the complete lesion group. Sham n = 19; incomplete lesion n = 13; complete lesion n = 28. For histological analysis sham n = 5; incomplete lesion n = 4; complete lesion n = 5. Data presented as mean ± SEM. ** *p* < 0.01, *** *p* < 0.001.

The apomorphine-induced turning test was performed at the end of the behavioral assessment to gain insight on the functional integrity of the dopaminergic system. This test revealed the identification of two cohorts within the 6-OHDA-lesioned group, one with marked turning behavior (complete lesion), the other without intense turning behavior (incomplete lesion) (Figure 1A). Statistical analysis revealed differences in apomorphine-induced turning behavior (H_2_ = 44.37; *p <* 0.001), with *post-hoc* analysis revealing a significantly higher number of rotations of the complete lesion group when compared to both incomplete lesion and sham groups (*p* < 0.05).

This analysis was further complemented by an assessment of tyrosine hydroxylase (TH)-immunohistochemistry (Figure 1B). The quantification of lesion extent performed through TH^+^ cells counts showed a marked degeneration in area A9 (F_2,11_ = 442.6, *p <* 0.0001) (Figure 1C). Further *post-hoc* analyses showed different degenerative profiles between sham animals and both 6-OHDA injected animals and between incomplete and complete lesion groups (*p* < 0.05), with incomplete lesion animals displaying ~70% degeneration in the injected side, compared to spared hemisphere and with complete lesion animals showing ~97% of TH^+^ cell loss (Figure 1C). In the case of area A10, a significant effect of 6-OHDA lesions was also found (F_2,12_ = 25.06, *p <* 0.0001) (Figure 1D). Both 6-OHDA injected animals displayed significant loss of TH^+^ cells when compared to controls (*p* < 0.05), with degeneration ranging from ~40% in animals with incomplete lesion to ~60% in the complete lesion group (Figure 1D).

### Motor performance after 6-OHDA lesions

**Figure 2:**
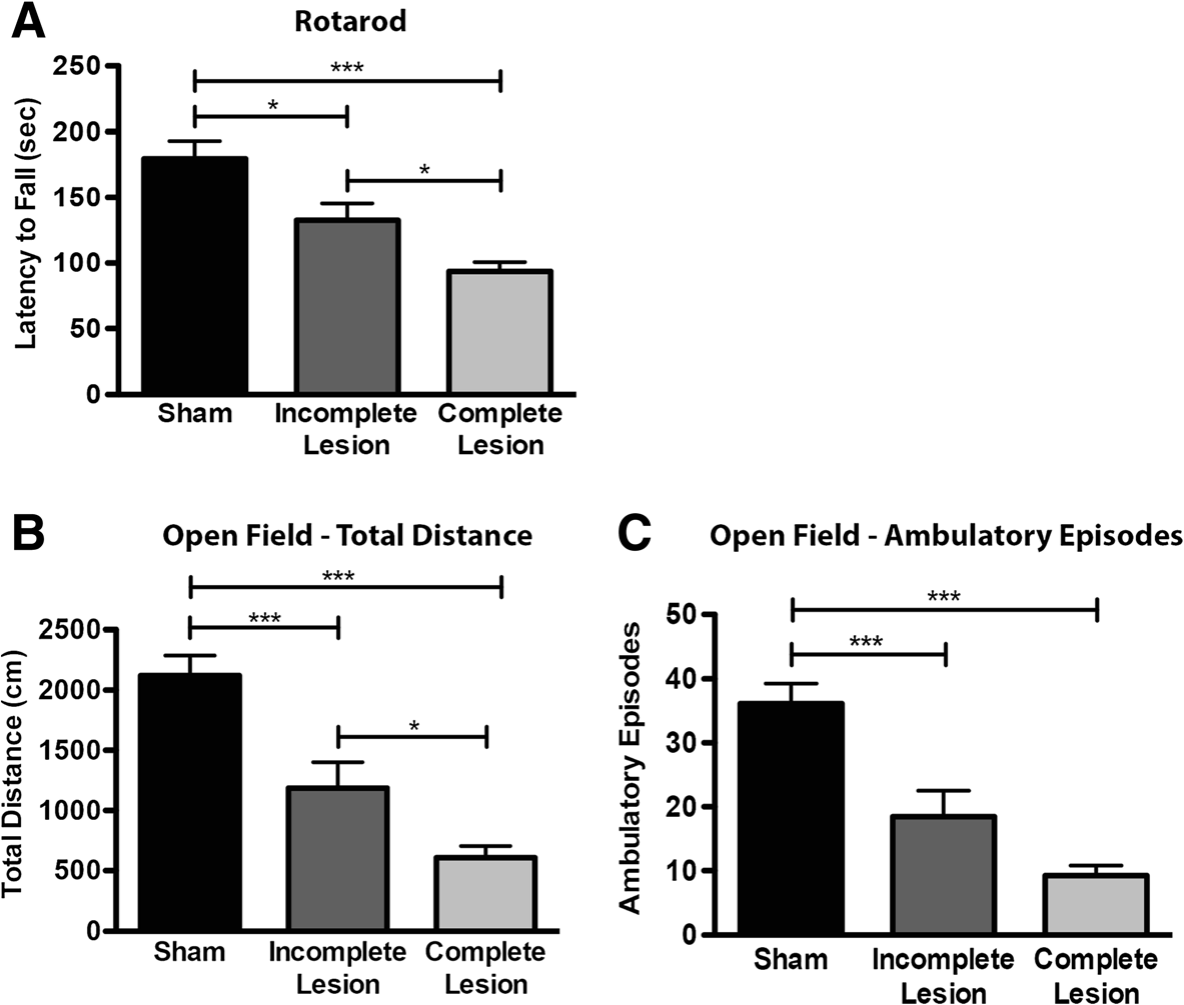
**Effect of unilateral dopaminergic degeneration on motor and exploratory behavior. A**) Unilateral 6-OHDA lesion caused a significant impairment in motor coordination on the rotarod test for both incomplete and complete lesion groups. **B**) On the open field arena both 6-OHDA groups display a reduction in total distance travelled. **C**) Overall reduction in movement initiation in the open field caused by 6-OHDA lesion. Data presented as mean ± SEM. * *p* < 0.05, *** *p* < 0.001. For rotarod sham n = 18, incomplete lesion n = 13, complete lesion n = 24. For open field sham n = 18, incomplete lesion n = 13, complete lesion n = 25.

Motor coordination, assessed in the rotarod (Figure 2A), was significantly impaired after lesions with 6-OHDA (F_2,52_ = 18.14, *p* < 0.0001). When compared to sham animals, both 6-OHDA-lesioned groups displayed clear motor coordination deficits, being, as expected, these changes greater in animals with complete lesions (*p* < 0.05).

Assessment of motor activity on the open field (Figure 2B) revealed an effect of 6-OHDA lesions on total distance travelled (F_2,50_ = 31.57, *p* < 0.0001), with both 6-OHDA-lesioned groups displaying a significantly lower motor activity than sham animals (*p* < 0.05); comparisons amongst the lesioned groups showed that the one with complete lesion was the most affected (*p* < 0.05). The effect of 6-OHDA lesions on movement initiation was also significant (F_2,50_ = 30.59, *p* < 0.0001) (Figure 2C). Comparisons between lesioned groups showed no differences; however animals in both these groups were clearly affected when compared to sham animals (*p* < 0.05).

### Effect of 6-OHDA lesions on anxiety and mood

**Figure 3:**
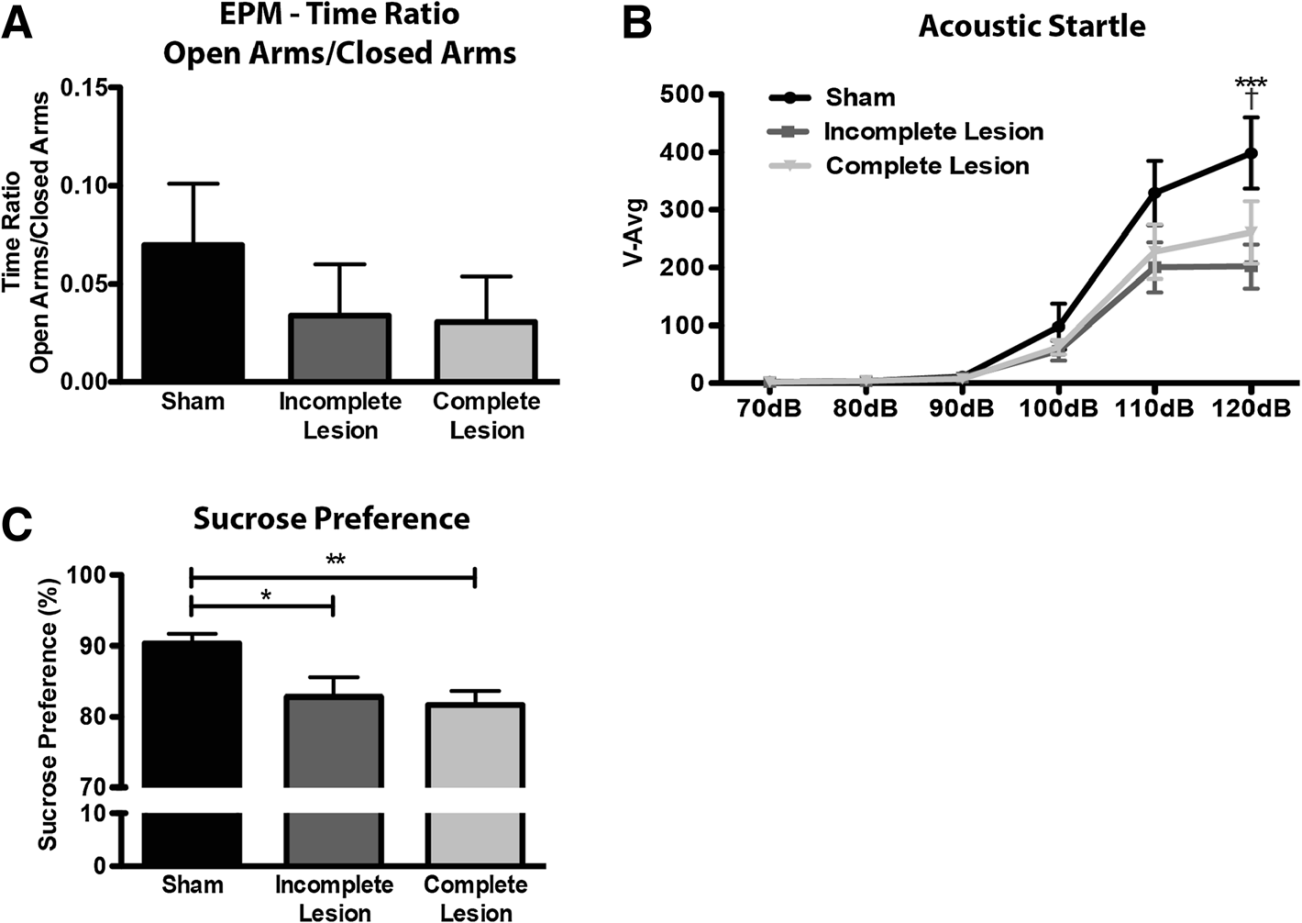
**Impact of 6-OHDA lesions on anxious and depressive-like behavior. A**) Related to sham animals incomplete and complete lesion groups show no signs for increased anxiety, as seen by open arms/closed arms time ratio on the elevated-plus maze (EPM). **B**) Assessment of acoustic startle response shows no signs of anxious-like behavior in both lesioned groups V-Avg = average startle response (arbitrary units). dB = decibels. **C**) Compared to sham animals, 6-OHDA lesions caused anhedonic behavior in the sucrose preference test in both incomplete and complete lesion groups Data presented as mean ± SEM. * *p* < 0.05, ** *p* < 0.01, *** *p* < 0.001, related to incomplete lesion, † *p* < 0.05, related to complete lesion. For EPM and acoustic startle sham n = 17; incomplete lesion n = 12; complete lesion n = 24. For sucrose preference test sham n = 17; incomplete lesion n = 12; complete lesion n = 28.

The elevated plus maze (EPM) paradigm was used to assess anxious-like behavior as a consequence of 6-OHDA lesions Figure 3A. Figure 3B concerns data of acoustic startle. No significant differences were found between the groups regarding the ratio between time spent on the open arms and time spent on the closed arms (H_2_ = 1.883, *p* = 0.39) (Figure 3A).

To overcome the influence of the motor component in the interpretation of EPM data, we used the acoustic startle response test as an additional measure of anxious-like behavior (Figure 3B). Again, there was no significant effect of 6-OHDA lesions in the startle response of the animals to auditory stimuli with increased intensity (F_2,245_ = 2.197, *p* = 0.12). Nonetheless, there was a significant effect of stimuli amplitude (F_5,245_ = 61.87, *p* < 0.0001) and of the amplitude × lesion interaction (F_10,245_ = 2.093, *p* = 0.025). Bonferroni *post-hoc* analysis showed a significantly lower startle response for both 6-OHDA lesioned groups at 120 dB stimuli, when compared to the sham group (*p* < 0.05).

Anhedonic behaviour, a key feature of depressive-like behaviour, was tested with the sucrose preference test (Figure 3C); importantly, this test is not significantly dependent on locomotor performance as the forced swimming test, the most commonly used test to assess depressive-like behavior. The preference for a sweet solution was shown to be significantly affected by 6-OHDA lesions (H_2_ = 11.11, p = 0.004), with both incomplete and complete lesion groups showing a robust decrease of preference when compared to sham animals (*p* < 0.05). These results suggest a depressive-like behavior after 6-OHDA lesions.

### Effects of 6-OHDA lesions in cognitive performance

**Figure 4:**
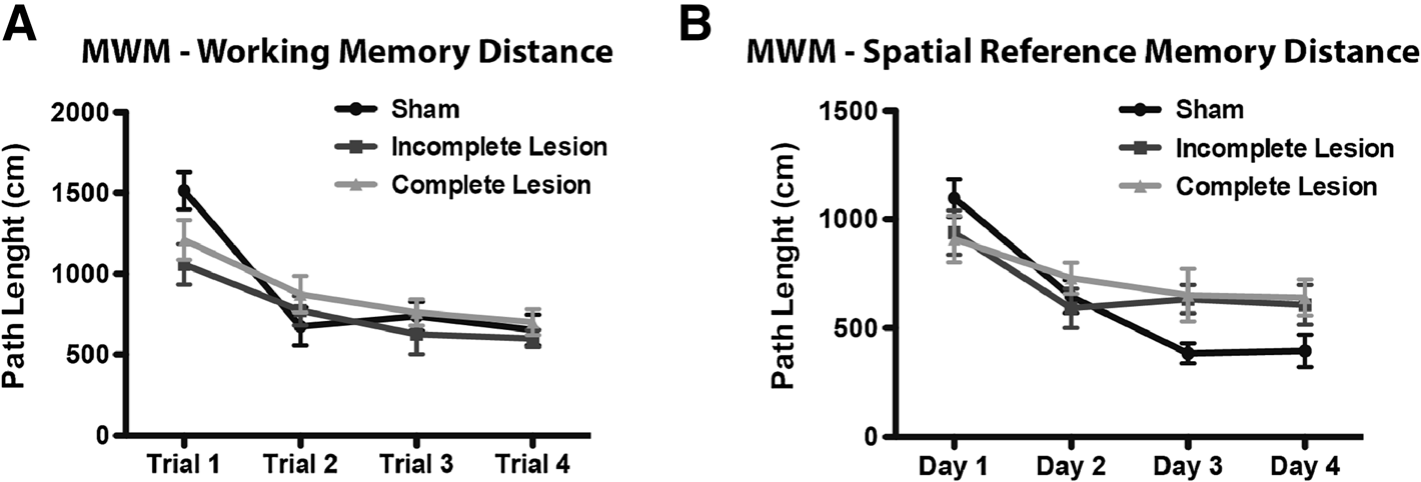
**Cognitive repercussions of unilateral dopaminergic degeneration. A**) Path length analysis in the working memory task shows no differences between both incomplete and complete lesion groups and the sham group. **B**) On the spatial reference memory task of MWM there were no differences in path length between sham animals and either incomplete and complete lesion groups. Sham n = 18; incomplete lesion n = 13; complete lesion n = 19. Data presented as mean ± SEM.

The cognitive function of 6-OHDA-lesioned animals was assessed by working and spatial reference memory performance in the Morris water maze test (MWM) (Figure 4). Analysis of path length during working memory task (Figure 4A), showed a significant effect of repeated sessions (F_3,138_ = 25.25, *p* < 0.0001), but no effect of lesion (F_2,128_ = 1.146, *p* = 0.3269) or interaction between the variables (F_6,138_ = 1.846, *p* = 0.0946). In the spatial reference memory task (Figure 4B) path length analysis showed no effect of lesion (F_2,135_ = 1.062, *p* = 0.3541). Nonetheless, there was a significant effect of repeated sessions (F_3,135_ = 19.25, *p* < 0.0001) and the interaction of the variables (F_6,135_ = 2.472, *p* = 0.0267). In other words, both reference and working memory performance were not affected by 6-OHDA lesions.

### Improving effects of levodopa and bupropion therapy in non-motor symptoms

**Figure 5:**
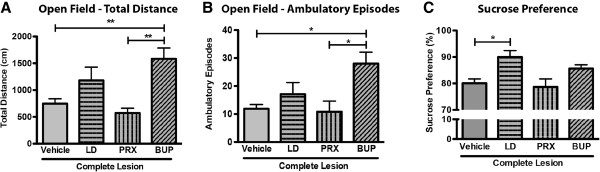
**Effect of dopaminergic and antidepressant therapy of animals with complete DA unilateral lesions. A**) On the open field arena bupropion (BUP) treatment significantly rescued spontaneous locomotion, whereas levodopa (LD) had a partial effect. Paroxetine (PRX) had no effect, related to vehicle-treated animals. **B**) BUP treatment improved motor initiation in the open field, while the effect of LD was partial. PRX had no effect when compared to vehicle treatment. **C**) Anhedonic behavior was significantly improved with LD treatment and partially by BUP, compared to vehicle-treated animals, while PRX showed no effect. Data presented as mean ± SEM. * *p* < 0.05, ** *p* < 0.01. Results from an additional set of animals distributed as following according to treatment: Vehicle n = 37; LD n = 10; PRX = Paroxetine n = 10; BUP = Bupropion n = 9.

To test whether the non-motor deficits displayed by animals with 6-OHDA lesions could be ameliorated, we administered LD (25 mg/kg, oral) and two different classes of antidepressants, PRX (6 mg/kg, i.p.) or BUP (10 mg/kg, i.p.) to complete lesioned animals. Exploratory behavior was assessed in the open field (Figure 5A); total distance travelled was significantly improved with treatment (F_3,56_ = 7.123, *p* = 0.0004). *Post-hoc* tests showed the efficiency of BUP in rescuing the deficits caused by complete 6-OHDA-induced lesions compared to animals treated with vehicle (*p* < 0.05), while in the case of LD its effect was only partial. PRX showed no improving action. The effect of treatment was also clear in movement initiation (H_3_ = 11.26, *p* = 0.0104) (Figure 5B), with BUP showing a significant improving action (*p* < 0.05) when compared to vehicle-treated animals, and LD only a modest effect. Again PRX had no positive effect.

In the case of anhedonic behavior (Figure 5C) there was a positive effect of treatment (H_3_ = 7.827, *p* = 0.049); *post-hoc* comparisons revealed that LD significantly rescued the decreased sucrose preference caused by 6-OHDA lesions (*p* < 0.05). Additionally, BUP treatment was found to have a partial (but not significant) effect in ameliorating anhedonic symptoms, while PRX showed no effect.

## Discussion

Our lesion protocol was successful in generating a rat model with lesions in both mesostriatal and mesocorticolimbic dopaminergic pathways. Interestingly, as shown by apomorphine-induced turning behavior, there were two distinct cohorts within the 6-OHDA-injected group, displaying different lesion extents. Animals displaying intense turning behavior, showed a nearly complete unilateral loss of TH^+^ neurons in the injected hemisphere, with losses reaching ~97% and ~60% in A9 and A10 regions, respectively. In turn, animals with moderate turning behavior displayed incomplete dopaminergic degeneration with a TH^+^ neuronal loss around 70% in area A9 and 40% in area A10. Remarkably, histological data revealed that regardless of lesion extent, neurons in A9 were consistently more affected than neurons in A10 in response to 6-OHDA, a result in accordance with reports supporting the higher sensitivity of neurons in A9 to dopaminergic insults [3,24,25].

Regarding motor function, we observed significant impairments in motor coordination in both lesioned groups, which were more severe in animals with more extensive lesions, in agreement with previous reports showing impaired coordination and skilled motor function in animals with DA lesion [21,26]. Besides deficits in motor coordination, lesioned animals also showed decreases in locomotor activity in the open field. As discussed elsewhere, locomotor activity in the open field depends on motor skills but also on the motivation to explore a novel arena [27]. While it is true that the rotarod data supports the idea that the reduced locomotion is, at least in part, influenced by motor impairments stemming from mesostriatal degeneration, it is also plausible that the lesion of the mesocorticolimbic network may contribute to a decreased drive to explore. Indeed, several preclinical and clinical studies support the role of this network, and associated frontostriatal projection areas, in motivational aspects of behavior [28-30]. Interestingly, this test also showed a lower number of ambulatory episodes in animals with 6-OHDA-induced DA lesions. This phenomenon may evoke the bradykinesia that characterizes PD, and is associated with mesostriatal dysfunction [6,31].

To further understand the behavioral implications of MFB dopaminergic lesions we assessed the presence of anxious-like behavior in this model. Whether anxiety is directly linked to DA degeneration or is a consequence of PD medication is still a matter of dispute [8,14]; however, anxiety symptoms are very common in PD patients and some authors state that anxiety in PD can be manifested even before the emergence of the first motor symptoms [14,32]. Curiously, experimental data in animal models of PD is also inconclusive. While a recent study showed no effect of bilateral MFB lesions on anxiety [33], previous studies had reported a significant anxiolytic effect of bilateral 6-OHDA lesions in the striatum [34], or even higher levels of anxiety in animals with either unilateral MFB lesions [35,36] or bilateral striatal lesions [37]. Our results on the EPM show no signs for the presence of anxiety-like behavior in animals with both incomplete and complete lesions. However, these animals also displayed reduced mobility in the maze (data not shown), which can confound the interpretation of the results. The assessment of acoustic startle responses was thus important to overcome the influence of the motor component on the EPM, as in this test the animals are not required to move. In line with the results on the EPM, the assessment of startle responses does not support the presence of anxious-like behavior, since both groups of animals with 6-OHDA lesions display even a decreased startle response to acoustic stimuli, when compared to sham-lesioned animals. On the light of these, but also previous findings, anxiety does not seem to be a common feature of this PD model.

On the spectrum of mood-related changes associated with human idiopathic PD, depression is also known to have a high prevalence, and to significantly affect the quality of life of patients [15,38]. It is widely accepted that dopamine is implicated in mood homeostasis [39] and, as for anxiety, depression can occur in PD patients even before the appearance of the first motor symptoms [40]. In the present study, we found that animals with either incomplete or complete unilateral DA denervation presented marked decrease in sucrose preference, indicative of depressive-like behavior. These results are in line with several studies in rodent models of PD [35,41-45]; interestingly, and fitting with the hypothesis that in human PD depression can emerge prior to motor disabilities, signs of depressive-like behavior were even described in models of pre-symptomatic stages of PD [34,37,46]. Bearing in mind the critical role of the mesocorticolimbic pathway in reward and hedonia, our results can be explained by the extent of dopaminergic denervation caused by 6-OHDA injections in this pathway. However, in contrast to the observed association between the extent of mesostriatal lesions and motor performance, our results indicate that the degree of mesocorticolimbic degeneration is not determinant for the magnitude of mood affection, namely anhedonic behavior. Importantly, the degeneration of mesostriatal neurons may also play a role in the development of these mood alterations as it was already proved that lesions in SNc could cause similar results [42,43]. However, the contribution of the mesostriatal pathway seems to be highly dependent on lesion site, and its effect upon projection areas, as lesions limited to the striatum failed to cause increased behavioral despair and anhedonia [34].

Several cognitive impairments, namely visuo-spatial, long-term and working memory deficits, set-shifting impairments, and ultimately dementia, are frequently associated with PD, as a result of mesocortical dopaminergic degeneration [10,47-50]. Accordingly, studies performed in animal models of PD showed how DA lesions were found to have a negative effect on cognitive performance [51-56]. However, in the present work, unilateral MFB lesions had no effect in any of the lesioned groups regarding both working and spatial reference memory function. We can hypothesize that our unilateral MFB lesions were not extensive enough to cause significant deficits on the tasks we assessed, and that only a dopaminergic depletion affecting both hemispheres will effectively reproduce cognitive impairments reminiscent of human PD [52-56].

After the behavioral characterization of our model, we decided to test whether the non-motor deficits observed in animals with complete lesions could be pharmacologically reverted; for that purpose we administered LD, to assess the effect of DA restoration, and two antidepressants, PRX and BUP (inhibitors of reuptake of serotonin and dopamine/norepinephrine, respectively). Compared to vehicle-treated animals, the administration of both LD and BUP improved exploratory behavior on the open field, particularly in the case of BUP. Improvements in this task are likely linked to some degree of motor rescue, combined with mood elevation leading to increased motivation to explore. In fact, we also noted a positive effect of both LD and BUP in hedonic behavior, given that both treatments increased the preference for sucrose, particularly LD. Even though the effect of both LD and BUP was not consistent in all behavioral measures and in some cases their action only partially rescued behavioral deficits, these results suggest that the non-motor changes found in animals with complete 6-OHDA-induced lesions are closely linked to changes in dopaminergic function. Clinical studies have shown how non-motor symptoms of PD are responsive to different dopaminergic treatments [57], and how LD is able to promote improvements in affective and apathetic symptomatology [12,58-60]. In animal models of PD, LD also proved to be beneficial for both mood and cognition [43,61]. In the case of BUP several reports have demonstrated how this drug has important stimulant properties, inducing hyperactivity in rodents on the open field [62-66]. This may help explaining the improvements we observed regarding exploratory activity. However, it should not be excluded the hypothesis that a mood improving action of BUP might also increase exploratory drive. In support of this hypothesis, our BUP treatment showed some potential to rescue anhedonic symptoms, and in fact BUP was already shown to be able to ameliorate depressive-like behavior [64,67]. Moreover, there is evidence proving the beneficial action of BUP in the treatment of depressive symptoms of PD [68,69].

Despite the relevance of DA network degeneration for the appearance of motor and non-motor symptoms in PD, the contribution of other neurotransmitters systems has also been the focus of several studies. For example, changes on the serotonergic system have also been described in the context of PD, and linked to the emergence of mood changes [9,70]. In animal models of PD the implication of changes in the serotonergic system and their relation with DA is not completely clear; in fact different studies found that DA lesions can either upregulate or downregulate serotonergic signaling [70]. Regardless of that, PRX, a common SSRI antidepressant, was recently shown to have positive action on mood in patients with PD [71], contradicting earlier reports [72]. Interestingly, our data suggests that in this particular model, behavioral deficits are unlikely related with serotonergic transmission, given that PRX administration was not successful in ameliorating any behavioral deficit. Changes in norepinephrine (NE) can further account for affective changes seen in PD [13]; and in line with this a combined serotonin/norepinephrine reuptake inhibitor was already shown to have mood improving actions in patients unresponsive to SSRI therapy [73]. Importantly, lesion protocols using 6-OHDA are known to affect NE transmission [37,74], which in the present study could play a role on the emergence of the mood changes reported. Given the additional role of BUP in inhibiting NE reuptake, we can hypothesize that its mood improving effect can be partially explained by the modulation of NE transmission, though further studies are needed.

## Conclusions

In summary, we showed that the unilaterally MFB 6-OHDA-lesioned model of PD presents a significant disruption in both mesostriatal and mesocorticolimbic dopaminergic pathways, with significant consequences on motor and some specific non-motor behaviors. Moreover, our results reveal that pharmacological manipulations of dopaminergic and noradrenergic signaling are efficient in rescuing many of these behavioral deficits, whereas serotonergic pathway does not seem so relevant in this context. This model can thus be used as a reliable tool to assess the effect of new treatments on multiple aspects of PD dysfunction and to test the efficiency of combined therapeutical approaches for PD, tackling both motor and non-motor features of the disease.

## Methods

### Animals and 6-OHDA Lesions

Eleven week old Wistar-Han male rats (Charles River, Barcelona) were housed, two per cage, under standard laboratory conditions: 12 hour light–dark cycle, 22°C room temperature, 55% relative humidity, food and water available *ad libitum*. All manipulations were done in accordance with the local regulations (European Union Directive 2010/63/EU) and NIH guidelines on animal care and experimentation.

Under ketamine-medetomidine (75 mg/kg: 0,5 mg/kg, i.p.) anesthesia the animals were placed on a stereotaxic frame with non-traumatic ear bars (Stoelting, USA), and unilaterally injected using an 30-gauge needle Hamilton syringe (Hamilton Company, Switzerland), with either vehicle (sham group, n = 19) or 6-OHDA hydrochloride (Sigma, USA) (6-OHDA group, n = 41) directly into the medial forebrain bundle (coordinates related to Bregma, AP = −4,4 mm; ML = −1,0 mm; DV = −7,8 mm; according to Paxinos and Watson [75]). At a rate of 1 μl/min, sham animals received 4 μl of 0,2 mg/ml ascorbic acid in 0,9% NaCl and 6-OHDA animals were injected with 4 μl 6-OHDA hydrochloride (3 μg/μl) with 0,2 mg/ml ascorbic acid in 0.9% NaCl. After injection the syringe was left in place for 4 minutes to allow diffusion. Behavioral assessment began three weeks after surgery.

### Behavioral assessment

#### Rotarod

Motor performance was evaluated on a Rotarod equipment (3376-4R; TSE Systems, USA), under an accelerating protocol previously described [21]. The first 3 days of testing served as training. The animals underwent a four trial test under an accelerating protocol going from 4 rpm to 40 rpm in 5 minutes, being allowed to rest for at least 20 minutes between trials. On the fourth day, using the same protocol, the latency to fall was recorded.

#### Open field

To assess exploratory activity, the animals were tested in the Open Field arena (ENV-515, Med Associates inc. USA) for 5 minutes, as previously described [76]. Total distance travelled and number of ambulatory episodes were recorded using a tracking software (SOF-811, Med Associates).

#### Elevated plus maze

To assess anxious-like behavior the animals were tested on an elevated plus maze (EPM) (ENV-560; Med Associates) as previously described [77]. Animals were placed in the central junction facing an open arm, and allowed to explore for 5 minutes. The test was recorded and the ratio between time in open arms and time in closed arms was measured.

#### Acoustic startle

Startle reflexes were measured in two identical startle response systems (SR-LAB, San Diego Instruments, USA) as previously described [77]. The animals were placed inside a ventilated, sound-attenuated chamber on a non-restrictive Plexiglas cylinder (i.d. 8.8 cm, length 22.2 cm). Cylinder movements were detected and measured by a piezoelectric element attached to the cylinder. Startle stimuli, presented through a high frequency speaker were sampled each millisecond (ms) during a period of 150 ms beginning at the onset of the startle stimulus. During this period average startle responses were recorded. Animals were habituated to the apparatus for 5 min, 24 hours before the test was performed. On the day of the test, after a 5 min habituation period each animal was presented five baseline startle stimuli (50-ms pulse of white noise at 120 dB) at an interstimulus interval of 30 s, followed by the random presentation of 52 startle stimuli, each with 50 ms in duration, and intensity between 70 to 120 dB, in 10-dB increments.

#### Sucrose preference test

To assess the presence of anhedonic behavior the animals were exposed to the sucrose preference test (SPT), adapted from Bessa et al. [78]. After 20 h of food and water deprivation the animals were placed in individual cages and presented with pre-weighted bottles, one containing tap water, the other one filled with 3% sucrose. Sucrose and water intake were measured as the difference between the initial and the final weight of the bottle. Sucrose preference was calculated according to the formula: sucrose preference = [sucrose intake/(sucrose intake + water intake)] × 100.

#### Morris water maze

To test cognitive function the animals were submitted to the Morris water maze test (MWM) as previously described [79]. Briefly, a black pool was divided into four quadrants, each associated with an extrinsic visual cue. The pool was filled with water (25°C), and a platform, invisible to the animals was submerged 1 cm below surface.

The working memory task was conducted during four days, with the animals performing four trials, 2 minutes each. Each trial had a different starting position. The trial ended either when the animal reached the platform or after 2 minutes. In the later case the animals were guided to the platform. The animals were allowed to rest in the platform for 20 seconds between each trial. In each of the four days the platform was hidden in a different position.

The spatial reference memory task was performed during four days with the first of them being the last of the working memory task. Experimental conditions were the same but with platform position kept constant for all the days. In both the working memory and spatial reference memory tasks the path length to reach the platform was recorded (View Track v.2.6., View Point Life Sciences inc., France). Animals showing persistent thigmotaxis or spending the entire test floating were excluded from the analysis.

#### Apomorphine turning behavior

To test apomorphine-induced turning behavior the animals received in the neck a subcutaneous injection of 0,05 mg/kg apomorphine hydrochloride (Sigma) dissolved in 1% ascorbic acid 0,9% NaCl, and placed on metal testing bowls (MED-RSS, Med Associates) for 45 minutes. The number of contralateral rotations was digitally recorded, and 6-OHDA-lesioned animals presenting more than 100 rotations in 45 minutes were considered to have a nearly complete lesion (n = 28), whereas the ones presenting less than 100 rotations were classified as having an incomplete lesion (n = 13). Sham animals (n = 19) showed no turning behavior.

### Histology

#### TH immunohistochemistry and lesion quantification

Animals were sacrificed with sodium pentobarbital, and transcardially perfused with a 4% paraformaldehyde in 0,1 M PBS. Mesencephalon coronal sections, 30 μm thick, were obtained with a vibratome (VT1000S, Leica, Germany). Four series of consecutive slices were obtained and one was processed for free-floating TH-immunohistochemistry. Briefly, slices were immersed for 20 minutes in 1 M PBS with 3% H_2_O_2_, followed by blocking for 2 hours with 5% fetal calf serum in 1 M PBS. Slices were then incubated over-night at 4°C with rabbit anti-mouse TH primary antibody (Millipore, USA, 1:2000 in 2% fetal calf serum in 1 M PBS), followed by incubation for 30 minutes with a biotinylated secondary anti-rabbit antibody (LabVision, USA), and 30 minutes incubation with an Avidine/Biotine complex (LabVision). Antigen visualization was performed with 3,3í-diaminobenzidine tetrahydrochloride (DAB, Sigma) (25 mg DAB in 50 ml Tris–HCl 0,05 M, pH 7,6 with 12,5 μl H_2_O_2_) and stopped at the desired time. The slices were mounted on superfrost slides and thionin counter-coloration was performed.

To assure a representative sampling between animals, six identical TH-labeled slices spanning the entire mesencephalon and including both A9 and A10 brain regions were selected in 5 animals per group. Using a bright-field microscope (BX51, Olympus, USA) equipped with a digital camera (PixeLINK PL-A622, CANIMPEX Enterprises Ldt., Canada), and with the help of Visiomorph™ software (V2.12.3.0, Visiopharm, Denmark) the boundaries of A9 and A10 areas were drawn. Delineation of regions of interest was performed through identification of anatomic reference points and with the help of rat brain atlas [75]. The A9 area included all the portions of SNc, whereas the A10 group included the ventral tegmental area, parabrachial nucleus, paranigral nucleus, rostral and caudal linear nuclei and interfascicular nucleus, as previously described [25]. Counting of total TH^+^ neurons in the areas drawn was performed on both hemispheres and the data presented as % of remaining TH^+^ neurons in the injected side, compared to the control side.

### Treatments

Treatments started two weeks before the behavioral assessment and continued until its end. Animals were treated daily with therapeutical doses of either paroxetine (PRX, n = 10) (6 mg/kg) or bupropion (BUP, n = 8) (10 mg/kg) dissolved in water. For levodopa (LD, n = 10) treatment, Sinemet pills (25 mg carbidopa/100 mg levodopa, Merck Sharp & Dohme, S.p.A, Italy) were smashed and suspended in tap water (5 mg/kg carbidopa + 25 mg/kg levodopa) and given orally 3 h before behavioral assessment. Behavior was then assessed between 3 and 4 h post-treatment, a therapeutical window with lower dyskinetic profile. An additional group (n = 37) received both i.p. injections and oral administration of water (Vehicle).

### Statistical analysis

All data was analyzed using the program GraphPad Prism 5 (GraphPad Software Inc., La Jolla, CA, USA). One-way ANOVA followed by Tuckey’s *post-hoc* test was used for analysis of data with Gaussian distribution. Non-gaussian data was analyzed with non-parametric analysis of variance (Kruskal-Wallis) followed by Dunn’s *post-hoc* test. Morris Water Maze and Acoustic Startle data were analyzed with repeated measures ANOVA, with time and acoustic intensity as within-group variable, respectively; followed by Bonferroni *post-hoc* test. All data shown has Mean ± S.E.M. Significance value set at *p <* 0.05.

## Abbreviations

6-OHDA: 6-hydroxydopamine; BUP: Bupropion; DA: Dopamine; EPM: Elevated plus maze; LD: Levodopa; MFB: Medial forebrain bundle; MWM: Morris water maze; NE: Norepinephrine; PD: Parkinson’s disease; PRX: Paroxetine; SPT: Sucrose preference test; SNc: Substantia nigra pars compacta; SSRI: Selective serotonin reuptake inhibitor; TH: Tyrosine hydroxylase.

## Competing interests

The authors declare that they have no competing interests.

## Authors’ contributions

MMC performed most of behavioral assessment, histological analysis, treatment protocols, data analysis and interpretation, and drafted the manuscript. FLC helped in surgical procedures and behavioral analysis. BC helped in treatment protocols and behavioral analysis. JMP helped in behavior assessment, data analysis and revision of the manuscript. CR and RL assisted in histological analysis and lesion quantification. AJR helped in data interpretation and in revision of the manuscript. NS and AJS contributed for data interpretation and for drafting, revision, and approval of the manuscript. All authors read and approved the final manuscript.

## Authors’ information

Nuno Sousa and António J Salgado, these authors share senior authorship.
